# Folivory has long‐term effects on sexual but not on asexual reproduction in woodland strawberry

**DOI:** 10.1002/ece3.4687

**Published:** 2018-11-14

**Authors:** Anne Muola, Johan A. Stenberg

**Affiliations:** ^1^ Department of Plant Protection Biology Swedish University of Agricultural Sciences Alnarp Sweden; ^2^ Section of Ecology, Department of Biology University of Turku Turku Finland

**Keywords:** clonal reproduction, compensation, fitness, *Fragaria vesca*, *Galerucella tenella*, leaf damage, plant tolerance, plant–herbivore interactions

## Abstract

Plant fitness is often a result of both sexual and asexual reproductive success and, in perennial plants, over several years. Folivory can affect both modes of reproduction. However, little is known about the effects of folivory on resource allocation to the two modes of reproduction simultaneously and across years. In a 2‐year common garden experiment, we examined the effects of different levels of folivory by the strawberry leaf beetle, *Galerucella tenella*, on current growth, as well as current and future sexual and asexual reproduction (runners) of perennial woodland strawberry, *Fragaria vesca*. In addition, we measured the chlorophyll content in leaves in the year of experimental damage to determine whether there was increased photosynthetic activity, and, thus, a compensatory response to herbivory. Finally, we tested whether the previous year's folivory, as a result of its effect on plant fitness, affected the level of natural herbivory the plant experienced during the subsequent year. In the year of experimental damage, plants that were exposed to moderate and high levels of folivory (25% and 50% leaf area consumed, respectively) increased their photosynthetic activity compared to control plants. However, only plants exposed to high folivory exhibited negative effects, with a lower probability of flowering compared to control plants, indicating that plants exposed to low or moderate folivory were able to compensate for the damage. Negative effects of folivory were carried over to the subsequent year. Plants that were exposed to moderate folivory (25% leaf area consumed) during first year produced fewer flowers and fruits in the subsequent year. Runner production was consistently unaffected by folivory. The effects of experimental folivory on the level of natural herbivory were mediated via its effects on plant fitness. Our results show that the negative effects of folivory only influence sexual reproduction in woodland strawberry. Furthermore, even though woodland strawberry can tolerate moderate amounts of folivory in the short term, the negative effects on fitness appear later; this highlights the importance of studying the effects of herbivory over consecutive years in perennial plants.

## 1 INTRODUCTION

One strategy that plants use to cope with herbivore damage is tolerance, that is, mechanisms that enable compensation for damage (Strauss & Agrawal, 1999; Tiffin, 2000). There are several known tolerance mechanisms, including increased photosynthetic activity, compensatory growth, activation of dormant meristems, changes in phenology, and the use of stored resources (reviewed in Tiffin, 2000). Whether or not plants are able to compensate for herbivore damage is known to depend on factors such as the timing and magnitude of the damage (e.g., Boege & Marquis, 2005; Garcia & Ehrlén, 2002; Hoque & Avila‐Sakar, 2015; Piippo, Hellström, Huhta, Rautio, & Tuomi, 2009; Ramula, 2008; Tiffin, 2002), plant life history (e.g., Boege & Marquis, 2005; Watson, 1995), the type of plant tissue that is damaged (e.g., Garcia & Ehrlén, 2002; Puentes & Ågren, 2012), and resource availability (reviewed by Wise & Abrahamson, 2007).

Many plant species reproduce both sexually and asexually, and both forms of reproduction contribute to plant fitness. Asexual fitness is most commonly a result of vegetative reproduction (i.e., clonal growth or vegetative propagation). However, most studies on tolerance have been conducted by concentrating on traits related to sexual fitness, such as number of flowers, fruits, or seeds (e.g., Carmona & Fornoni, 2013; Muola, Mutikainen, Laukkanen, Lilley, & Leimu, 2010; Puentes & Ågren, 2012); thus, less information is available pertaining to long‐lived plants employing both sexual and asexual reproduction. Because sexual and asexual reproduction often occur simultaneously, an allocation trade‐off may exist between the two modes of reproduction (Abrahamson, 1980). Given that herbivore consumption can directly damage plant meristems and alter the availability of resources, it could also affect the balance and the potential allocation trade‐offs between sexual and asexual reproduction. Compared to asexual reproduction, sex increases and maintains genetic variation through recombination, and, thus, is likely to promote adaptations; such traits may include those that function as defence against herbivores (Hamilton, Axelrod, & Tanese, 1990; Johnson, Smith, & Rausher, 2009). However, sexual reproduction is, in general, considered to be more costly than asexual reproduction. In contrast, asexual reproduction has several ecological advantages and most of them can be seen as beneficial when the plant faces damage by herbivores. These advantages include persistence in habitats unfavorable for sexual reproduction, the ability of clones to forage for resources in heterogeneous environments, and opportunities to spread the risk of death among ramets (Caraco & Kelly, 1991; Stuefer, DeKroon, & During, 1996).

In addition to the complexity added by dual modes of reproduction, it is known that herbivory can have both short‐term and long‐term effects on fitness (Ehrlén, 2002; Muola et al., 2010; Puentes & Ågren, 2012; Strauss, 1991). While short‐term effects are well documented, long‐term effects are less well understood (but see Huhta, Rautio, Hellström, Saari, & Tuomi, 2009; Muola et al., 2010; Puentes & Ågren, 2012; Vallius & Salonen, 2000). Metabolic compensation for herbivore damage may force plants to increase their use of stored resources or reduce their ability to store resources for future use, which may, in turn, defer the negative effects on fitness until subsequent growing seasons (Huhta et al., 2009). Furthermore, in perennial plants, the negative effects of herbivore damage on current and/or future reproduction may be confounded by the costs of reproduction (Venecz & Aarssen, 1998). For instance, if herbivore damage inhibits current reproduction, resources that would have been invested in reproduction may instead be stored and invested in reproduction in the following year. Thus, damaged plants could, using stored resources, achieve higher fitness in the subsequent year compared to undamaged plants. Alternatively, if damaged plants immediately regrow and reproduce to mitigate the costs of herbivore damage on current reproduction, this investment may reduce future fecundity as a consequence of the costs of compensatory reproduction. In addition to more direct effects on plant performance and fitness, herbivory during 1 year might at least partially determine the probability or amount of herbivory during subsequent years. For instance, many herbivores prefer larger or more vigorous plants (Price, 1991; Schlinkert et al., 2015). Plants that are negatively affected by herbivory are likely to be less vigorous compared to undamaged plants. Even though the amount of herbivory that plants experience is affected by many other factors than plant vigor, herbivores’ preference to feed on vigorous plants is likely to add to the long‐term effects of herbivory and potentially also to the plants’ ability to compensate for the herbivore damage. Given these potential long‐term impacts, it is important to study the effects of herbivore damage over several years in perennial plants.

In this study, we measured the ability of woodland strawberry (*Fragaria vesca*) to tolerate leaf damage by the strawberry leaf beetle *Galerucella tenella* (Coleoptera: Chrysomelidae), hereafter strawberry leaf beetle (SLB). This was done by measuring the effects of different levels of leaf damage on survival, growth, and chlorophyll content, as well as sexual and asexual reproduction of woodland strawberry across two years. Nothing is yet known about the effects of leaf feeding although florivory by SLB has previously been shown to have negative effects on the sexual fitness of woodland strawberry (Muola et al., 2017). Woodland strawberry is a perennial herb that reproduces both sexually and asexually through the production of runners (hereafter clonal reproduction; Angevine, 1983). Flower initiation of woodland strawberry occurs in the autumn (Heide & Sønsteby, 2007); therefore, resources lost to herbivores may affect the plant's ability to initialize flowers and, thus, flowering in the subsequent growing season. Our main question was as follows: How much damage is woodland strawberry able to tolerate without negative effects on current and future growth, survival, chlorophyll content, and reproduction? We expected low to moderate amounts of damage to be readily tolerated, while high amounts of damage are likely to have negative effects over subsequent years. In addition, we were able to investigate whether damage in 1 year affects the level of natural herbivory that the plants experience the following year.

## 2 MATERIAL AND METHODS

### 2.1 Study species

Woodland strawberry, *F. vesca* (Rosaceae), is a perennial plant that occurs throughout the Northern Hemisphere, growing in various habitats such as forest clearings, forest edges, roadsides, and paths (Hancock, 1999). Flower initiation is controlled by temperature and photoperiod and occurs in the autumn (Heide & Sønsteby, 2007). In the study area, the initiated flower buds develop in the following spring, and the main flowering period lasts from late May to early July. Flowers are hermaphrodite and self‐compatible to various degrees (Egan, Muola, & Stenberg, 2018; Angevine, 1983). Woodland strawberry is insect pollinated, and wild plants reproduce both sexually and clonally through formation of aboveground runners (Hancock, 1999).

**Figure 1:**
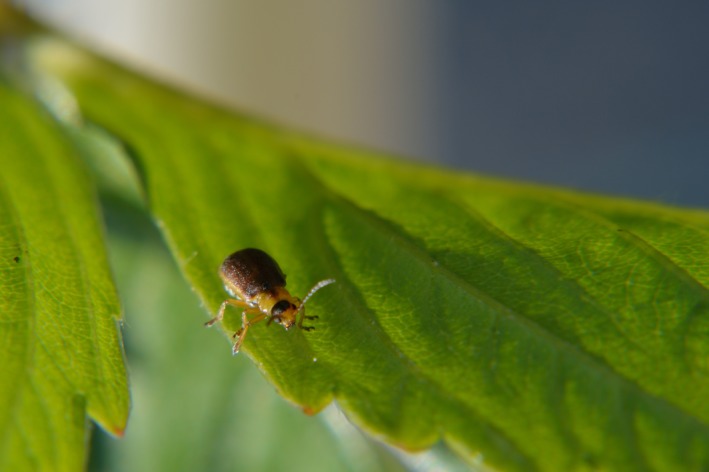
Strawberry leaf beetle, *Galerucella tenella*, on a woodland strawberry leaf. Photograph by Alejandro Ruete

The strawberry leaf beetle (SLB), *G. tenella* L. (Coleoptera, Chrysomelidae), is a common oligophagous herbivore that feeds on many species of Rosaceae (Olofsson & Pettersson, 1992; Stenberg & Axelsson, 2008; Figure 1). Adult SLBs overwinter in the soil and emerge in April to May. They forage on both leaves and flowers and can eat petals, seeds, and fruits. Oviposition starts in late May to early June and eggs are laid on the leaves. Eggs hatch in June to July. Larvae feed for 2–4 weeks on aboveground tissues before pupating in the ground. After approximately eight days, the pupae hatch and the adults that emerge feed on leaves before overwintering from mid‐September (Olofsson & Pettersson, 1992; Stenberg, Witzell, & Ericson, 2006). All SLB individuals used in the experiment were collected from a natural population close to SLU Ultuna campus (N59.810°, E17.667°) during late April and early May 2013.

### 2.2 Experimental design

To determine the effects of SLB damage on survival, growth, chlorophyll content, and sexual and clonal reproduction of woodland strawberry, we conducted a 2‐year common garden experiment. Experimental plants were divided into four treatment groups (described below) representing different damage levels: control, 10%, 25%, and 50% leaf area consumed by SLB.

The woodland strawberry genotypes (maternal plants of the experimental plants) were collected in spring 2012 from 70 geographically distinct locations across Uppsala County, Sweden (N59.858°, E17.650°). The minimum distance between locations was ten kilometers. Uppsala County is 8,209 km^2^ in area, and the 70 locations were randomly selected within this area. At the SLU Ultuna campus, plant genotypes were cloned by means of runners for seven to eight vegetative generations to minimize potential maternal effects. In May 2013, four plants per genotype, altogether 280 plant individuals, were replanted outside into 0.33‐L pots containing Hasselfors™ (Hasselfors Garden, Örebro, Sweden) potting medium. All experimental plants were placed outside on three separate tables (hereafter blocks) in a caged area at the SLU Ultuna campus (N 59°49.025′, E 17°39.451′). Temperatures and weather conditions in the caged area followed outdoor temperatures and conditions. Wild pollinating insects were able to enter the area, but the mesh (diameter: 20 × 20 mm) excluded birds that could consume the ripened fruits. One plant per genotype was randomly assigned to one of four damage treatments (*n* = 70 per treatment) representing different damage levels: control, 10%, 25%, and 50% leaf area consumed by SLB. In June 2013, each experimental plant was enclosed in a perforated (hole diameter 0.5 mm) polythene bag (Baumann Saatzuchtbedarf, Waldenburg, Germany). To generate the herbivore damage, two beetles, one female and one male, were placed inside the bag and they were allowed to feed until the required damage level was reached (maximum eight days). To score the damage level, plants were monitored daily and the damage level was estimated visually (by eye). The used herbivore density corresponds to natural beetle densities found in a previous study (Stenberg, 2014). Due to an unfortunate human error, some of the experimental plants were sprayed with pesticides and had to be excluded from the experiment. After excluding the plants that had been exposed to pesticides, we ended up with a total of 186 plants and the following number of replicates in different treatments: control (*n* = 66), 10% (*n* = 36), 25% (*n* = 52), and 50% (*n* = 32) leaf area consumed by SLB.

To measure the effect of SLB leaf damage on growth, chlorophyll content, and sexual and clonal reproduction of woodland strawberry, we determined the number of leaves prior to the damage treatment in June 2013 and 4 weeks after the damage treatment in July 2013. We calculated the number of flowers and fruits, and recorded whether each plant was producing runners (i.e., reproducing clonally) in July and early September 2013. Given that increased photosynthetic activity following herbivore damage is one of the most widely acknowledged mechanisms of tolerance (Rosenthal & Kotanen, 1994; Strauss & Agrawal, 1999; Tiffin, 2000), we measured the foliar chlorophyll content as an estimate of compensatory response to SLB damage. We used a handheld optical chlorophyll meter (SPAD‐502 Plus, Konica Minolta, Japan) and took two measurements on averaged aged and sized leaves on each plant and calculated the mean value (SPAD value). Measurements with SPAD‐502 meters produce relative values that are proportional to the amount of chlorophyll in the leaf (Guler, Macit, Koc, & Ibrikci, 2006; Ling, Huang, & Jarvis, 2011). For the purpose of this study, we were only interested in the relative differences between the plants and did not convert the values to absolute units.

In order to determine the effect of damage on plant survival, the experimental plants were planted in sandy soil in an open agricultural field in Krusenberg (N59.741°, E17.684°) 15 km south of Uppsala, Sweden, in September 2013. The plants were planted following the same block design as in the caged area (see above), that is, each experimental plant belonged to the same block in the caged area and in the field, but the position within the block was again randomized in the field. The distance between the plants was 50 cm, and the entire common garden was covered with MyPex^®^ Groundcover before planting in order to reduce weed densities. No irrigation or fertilizer was used.

To determine whether the leaf damage by SLB affects survival and sexual and clonal reproduction of woodland strawberry in the following year, we recorded whether plants had survived the winter and started to grow in early May 2014. In addition, we determined the number of flowers at the beginning and end of June 2014, and the number of fruits at the end of June and early July 2014. In early September 2014, we collected all runners. The runners were dried at room temperature (20°C) for 4 weeks and stored for an additional 3 months, after which they were weighed. Runner biomass was used as a measure of asexual reproduction potential.

Experimental plants growing in the Krusenberg common garden were subject to natural leaf herbivory during 2014. Thus, to test the potential effect of the previous year's experimental damage—via its potential effects on plant performance and fitness—on natural herbivory, we conducted a census to record damage caused by naturally occurring herbivores in June 2014. Most of the damage was caused by SLB. In addition, leaf‐feeding lepidopterans *Ceramica pisi* (Noctuidae) and *Cnephasia interjectana* (Tortricidae) larvae were observed on experimental plants. *C. pisi* is a generalist moth that feeds on genera including *Rubus* and *Salix* (Robinson, Ackery, Kitching, Beccaloni, & Hernández, 2010). *C. interjectana* is a pest of cultivated garden strawberry (Cross et al., 2001). We assessed the total amount of damage each plant had experienced, meaning that we did not quantify damage by different herbivores separately. According to the amount of damage, plants were classified into one of four classes: (a) 1% or less of leaf area damaged, (b) 5% or less of leaf area damaged, (c) 5% to 20% of leaf area damaged, and (d) 20% or more of leaf area damaged.

### 2.3 Statistical analysis

#### 2.3.1 Year of experimental damage

We analyzed the effect of leaf damage on plant growth and chlorophyll content in the year of experimental damage using analyses of covariance (PROC GLIMMIX, SAS 9.4). Plant growth was measured in terms of number of leaves and chlorophyll content as a SPAD‐value. In the model, damage treatment and block were used as fixed factors and plant size (number of leaves) before damage treatment was included as a covariate to control for the effect of initial size. The normality and equality of variances of the residuals were assessed by visual examination and Levene's test, respectively.

Only 56% of experimental plants flowered, 43% of the flowering individuals set fruit, and 68% of all experimental plants produced runners in the year of the experimental damage. Thus, the distributions of estimates measuring sexual and clonal reproduction were naturally binomial. Due to this, we tested the effect of damage on plant reproduction by modeling the probability that a plant would flower (flowering vs. not flowering), set fruits (fruits vs. no fruits), and reproduce clonally (runners vs. no runners) with three separate generalized linear models for binomial data. We included only individuals that were flowering in the analysis of fruit setting, and, thus, the binomial response variable “fruits versus no fruits” is comparable with fruit set (number of fruits per flower; see below “Year following experimental damage”). Damage treatment and block were included as fixed factors, and plant size (number of leaves) before experimental damage was included as a covariate to control for the effect of plant size on reproduction. We used a generalized linear model (PROC GLIMMIX, SAS 9.4) with logit link function.

#### 2.3.2 Year following experimental damage

Given that all the experimental plants survived transplantation to the Krusenberg common garden in September 2013, we did not test the effect of experimental damage on survival. In the year following experimental damage, 96% of the experimental plants flowered and all plants produced runners. Thus, to analyze the effect of the previous year's experimental damage on sexual and clonal reproduction, we constructed three separate analyses of covariance with number of flowers, fruit set, and runner biomass as response variables, and one generalized linear model with number of fruits as the response variable (PROC GLIMMIX, SAS 9.4). While number of fruits is a more direct measure of fitness, fruit set (i.e., number of fruits per flower) describes the ability of flowers to develop into fruits. Previous year's experimental damage and block were used as fixed factors. In addition, we included initial plant size (measured as number of leaves before previous year's experimental damage treatment) as a covariate in all the models to control for the effect of initial plant size. Due to missing information, we had data from 180 plant individuals in the model of the effect of previous year's experimental damage on the number of flowers and from 174 plant individuals in the model of the effect of previous year's experimental damage on the runner biomass. In addition, we excluded plant individuals that did not flower the year following the experimental damage from the models relating to the effect of the previous year's experimental damage on number of fruits and fruit set. Thus, in the models testing the effect of the previous year's experimental damage on the number of fruits and fruit set, we had data from 174 and 173 plant individuals, respectively. For the analyses of covariance (number of flowers, fruit set, and runner biomass), the normality and equality of variances of the residuals were determined by visual examination and Levene's test, respectively. We used a negative binomial distribution and log link function in the model testing for the effect of experimental damage on the number of fruits.

**Figure 2:**
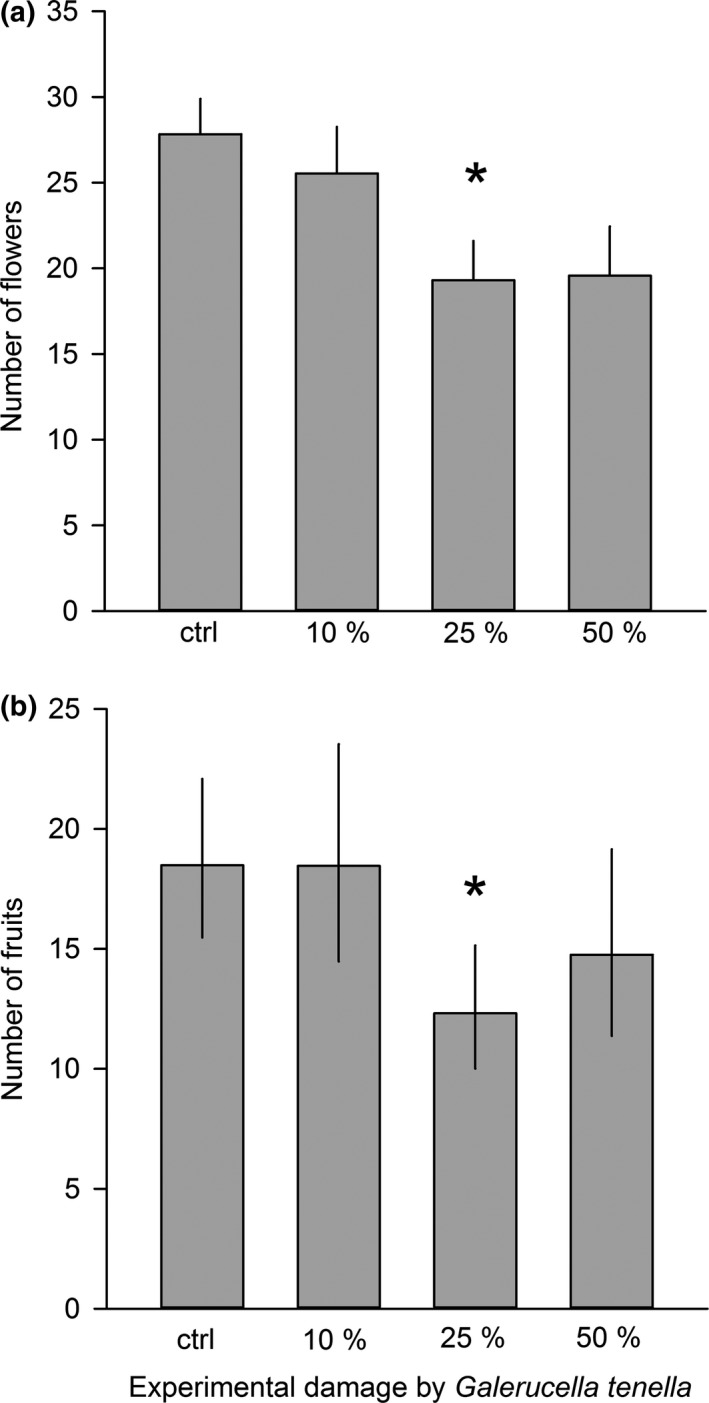
The effect of *Galerucella tenella* (SLB) damage on (a) number of flowers and (b) number of fruits in the year following experimental damage (LSMEANS ± SE). Statistical significance between control and different damage levels was obtained using a priori contrasts and is denoted with an asterisk. *p < 0.05

To investigate whether previous year's experimental herbivore damage, as a result of its effects on plant fitness, affects the level of natural herbivory a plant experiences during the following year, we tested the association between natural herbivory and the number of flowers. We calculated the correlation coefficient for number of flowers against natural damage using Spearman's partial correlation in order to remove the potential effect of block on natural herbivory. Because number of flowers was negatively affected by the experimental damage in the year before (see Results, Table 2 and Figure 2b), it was used as a measure of plant fitness. Furthermore, number of flowers is positively correlated with other plant performance‐ or fitness‐related traits such as plant size, number of fruits, and runner biomass (data not shown).

In all the models, interactions with covariate and explanatory variables were tested and nonsignificant interactions were removed from the final models. Block was included to eliminate the potential effects of environmental variation due to the different positions of experimental plants first in the caged area and, later, in the common garden in Krusenberg. Block was treated as a fixed factor since we had only three blocks. Differences between control plants and plants that experienced different amounts of SLB damage were examined with pairwise a priori contrasts, specified as control versus each damage level in all the analyses where the main effect of controlled damage treatment was statistically significant. Bonferroni correction was used to correct for multiple comparisons. All analyses were conducted with SAS 9.4 (SAS Enterprise Guide 6.1/SAS 9.4 Cary, NC).

## 3 RESULTS

### 3.1 Year of experimental damage

**Figure 3:**
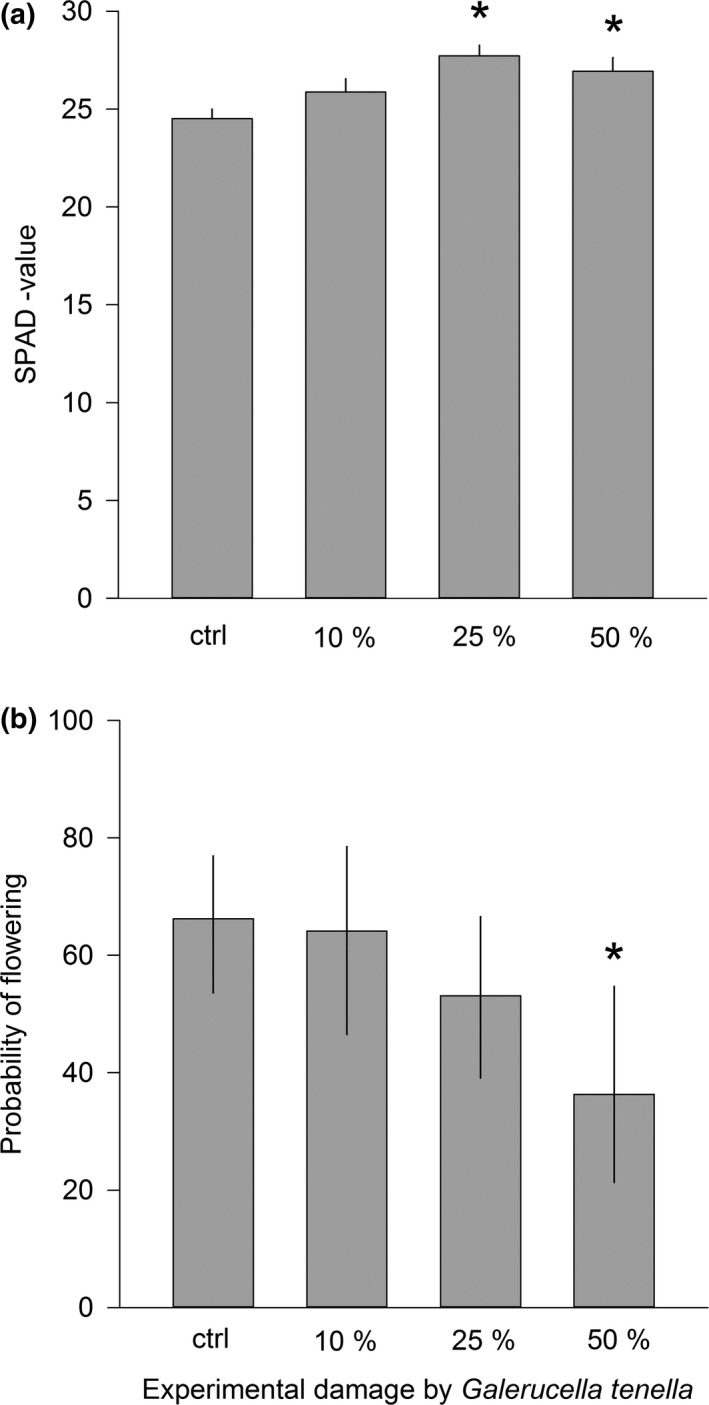
The effect of *Galerucella tenella* (SLB) damage on (a) chlorophyll content measured as SPAD value and (b) the probability of flowering during the year that experimental damage was inflicted (LSMEANS ± SE). Plant size was measured as number of leaves. Statistical significance between control and different damage levels was determined using a priori contrasts and is denoted with an asterisk. *p < 0.05

Plants with 25% and 50% leaf damage had 12% and 9% higher chlorophyll content than control plants (contrasts: ctrl vs. 25% damage *F*_1, 177_ = 19.45, Bonferroni corrected *p* < 0.0001; ctrl vs. 50% damage *F*_1, 177_ = 8.28, Bonferroni corrected *p* = 0.0135; Table 1, Figure 3a) indicating increased photosynthesis as a compensatory response to the herbivory, and, accordingly, SLB damage did not affect plant growth measured as number of leaves (Table 1). During the year of damage, only 56% of experimental plants flowered producing on average 12.9 ± 11.9 flowers (mean ± *SD*). The probability of flowering was significantly lower only for plants with 50% leaf damage compared to control plants (contrast: *F*_1, 179_ = 7.19, Bonferroni corrected *p* = 0.0240; Table 1, Figure 3b). In addition, plant size affected the probability of flowering (Table 1): Flowering individuals were, on average, larger (initial size 6.0 ± 0.2 leaves) than those that did not flower (initial size 5.1 ± 0.2 leaves). Out of 105 flowering individuals, 45 produced fruits in the year of the damage treatment. On average, fruit‐producing plants produced 5.1 ± 4.3 fruits (mean ± *SD*). Neither damage treatment nor plant size affected the probability of setting fruits or the probability of producing runners (probability to set fruits: control 29.0 [16.8, 45.3; mean (SE lower, upper)], 10% leaf area consumed 57.8 [36.4, 76.6], 25% leaf area consumed 43.5 [25.9, 62.9], and 50% leaf area consumed 45.3 [19.2, 74.3], and probability to produce runners: control 66.7 [54.2, 77.1; mean (SE lower, upper)], 10% leaf area consumed 63.2 [46.2, 77.4], 25% leaf area consumed 68.2 [54.2, 79.5], and 50% leaf area consumed 81.2 [63.6, 91.4]; Table 1). Runner producing plants had on average 7.9 ± 9.5 (mean ± *SD*) runners.

**Table 1:** The results of the analyses of covariance testing the effects of experimental damage (damage: control, 10%, 25%, and 50% leaf area consumed by *Galerucella tenella* [SLB]), initial plant size (covariate), and block on plant size and chlorophyll content (SPAD value), and generalized linear models testing the effects of experimental damage (damage), initial plant size (covariate), and block on the probability of flowering, setting fruits, and producing runners during the year of the experimental damage

Dependent variable	Source of variation	*df*	*F*	*p*
Plant size (number of leaves)	Damage	3, 177	1.69	0.1700
	Covariate (initial plant size)	1, 177	76.32	**<0.0001**
	Block	2, 177	3.20	**0.0433**
Chlorophyll content (SPAD value)	Damage	3, 177	7.12	**0.0002**
	Covariate (initial plant size)	1, 177	1.20	0.2744
	Block	2, 177	8.87	**0.0002**
Probability of flowering	Damage	3, 179	2.73	**0.0456**
	Covariate (initial plant size)	1, 179	7.54	**0.0066**
	Block	2, 179	0.04	0.9609
Probability of setting fruits	Damage	3, 98	1.59	0.1976
	Covariate (initial plant size)	1, 98	0.00	0.9841
	Block	2, 98	2.94	0.0575
Probability of producing runners	Damage	3, 179	0.94	0.4241
	Covariate (initial plant size)	1, 179	0.29	0.5883
	Block	2, 179	0.91	0.4028

Bold value denotes *p*‐values of < 0.05.

### 3.2 Year following experimental damage

Strawberry leaf beetle damage had a negative effect on flower and fruit production in the year following experimental damage (Table 2, Figure 2). Compared to control plants, plants with 25% of their leaf area consumed had significant, and plants with 50% of their leaf area consumed nearly significant, reduction in their flower production during the year following experimental damage. The reduction in flower production was 31% for plants that had 25% of their leaf area consumed (contrast: *F*_1, 173_ = 7.65, Bonferroni corrected *p* = 0.0189; Figure 2a) and 30% for plants that had 50% of their leaf area consumed (contrast: *F*_1, 173_ = 5.52, Bonferroni corrected *p* = 0.0597). The reduction in fruit production was largest (33%) for plants that had 25% of their leaf area consumed (contrast: *F*_1, 167_ = 8.67, Bonferroni corrected *p* = 0.0111; Figure 2b). The previous year's experimental damage did not affect fruit set (control 0.679 ± 0.027 [mean ±SE], 10% leaf area consumed 0.682 ± 0.036, 25% leaf area consumed 0.610 ± 0.030%, and 50% leaf area consumed 0.643 ± 0.038; Table 2). Initial plant size affected both the number of flowers and the number of fruits but not fruit set: Plants that were larger to begin with produced, on average, more flowers and fruits even during the following growing season (data not shown; Table 2). Experimental damage did not affect runner production during the following year (control 22.3 ± 1.1 g [mean ± SE], 10% leaf area consumed 23.5 ± 1.6 g, 25% leaf area consumed 21.9 ± 1.3 g, and 50% leaf area consumed 18.5 ± 1.7 g; Table 2). We found a positive correlation between natural herbivory and the number of flowers, indicating that herbivores preferred plants with more flowers (*r* = 0.3585, *n* = 180, *p*<0.0001).

**Table 2:** The results of the analyses of covariance testing the effects of previous year's experimental damage (damage treatment) and block on number of flowers, fruit set, and runner biomass, and generalized linear model for number of fruits. Initial plant size was used as a covariate in all models

Dependent variable	Source of variation	*df*	*F*	*p*
Number of flowers	Damage treatment	3, 173	3.43	**0.0183**
	Block	3, 173	7.95	**0.0005**
	Covariate (initial plant size)	3, 173	5.58	**0.0193**
Number of fruits	Damage treatment	3, 167	3.53	**0.0161**
	Block	2, 167	13.1	**0.0001**
	Covariate (initial plant size)	1, 167	6.08	**0.0147**
Fruit set	Damage treatment	3, 166	1.24	0.2962
	Block	2, 166	2.99	0.0528
	Covariate (initial plant size)	1, 166	0.29	0.5906
Runner biomass	Damage treatment	3, 168	1.63	0.1836
	Block	2, 168	2.05	0.1314
	Covariate (initial plant size)	1, 168	1.00	0.3181

Bold value denotes *p*‐values of < 0.05.

## 4 DISCUSSION

We found that damaged plants increased their chlorophyll content, thereby compensating for folivory. As a result, only plants with large amounts of leaf damage experienced negative effects on their sexual reproduction in the year of experimental damage. Given that most resistance traits reduce but do not completely eliminate damage, even relatively well‐defended long‐lived perennials often suffer severe biomass losses to herbivory (Haukioja & Koricheva, 2000; Turcotte, Turley, & Johnson, 2014). Thus, the long life span and the long exposure time to herbivores may favor the evolution of tolerance in perennial plants (Haukioja & Koricheva, 2000). Interestingly, in the year following experimental damage, negative consequences for flower and fruit production were more pronounced for plants with moderate (25%) damage than for plants with high (50%) damage. Our findings indicate that although woodland strawberry seems able to tolerate moderate amounts of leaf damage relatively well in the short term, the negative effects of folivory appear later and are specifically focused on sexual reproduction. Given these findings, studying only the short‐term effects of folivory in perennial plants may lead to misleading conclusions. Thus, the effects of herbivory are important to study over longer time periods in perennial plants.

The ability to compensate for herbivore damage is associated with the potential for regrowth following defoliation (Stowe, Marquis, Hochwender, & Simms, 2000). Regrowth is dependent on resource availability and allocation patterns, plant architecture, and phenology (Stowe et al., 2000; Strauss & Agrawal, 1999; Tiffin, 2000). Indeed, our results suggest that plants that experienced less than 50% damage were able to compensate for the lost tissues without compromising the resources available for reproduction during the year of experimental damage, indicating that SLB damage is tolerated relatively well in the short term. However, in the year after experimental damage, we observed negative consequences for flower production especially in plants that had experienced 25% experimental leaf damage. In addition, in the year following the experimental damage, fruit production was negatively affected in plants that experienced moderate (25%) leaf damage during the previous year. One reason underlying this finding could be that plants that experienced most damage suppressed further sexual reproduction and remained vegetative during the ongoing growing season and, thus, stored resources for future growth and reproduction (Huhta et al., 2009; Strauss, 1991; Venecz & Aarssen, 1998). Plants that experienced only 25% leaf damage responded differently. In the year of damage, their growth, and sexual and clonal reproduction did not differ significantly from those of control plants. However, since strawberry flowers are initiated during the previous growing season (Heide & Sønsteby, 2007), it is likely that, due to compensating for herbivore damage without compromising the current sexual reproduction, plants with only 25% damage had fewer available resources to initiate flower production and this delayed the negative fitness effects of SLB damage until the following growing season. The observed carry‐over effect of leaf damage is probably due to differences in resource reallocation patterns after different amounts of damage, and it may be further confounded by the potential costs of (sexual) reproduction (Primack, Miao, & Becker, 1994; Puentes & Ågren, 2012; Vallius & Salonen, 2000). Our findings emphasize the importance of following the effects of herbivore damage over more than one growing season in perennial plants. In addition, we found that even if leaf damage decreased the probability of flowering and the number of flowers and fruits, it did not affect the probability of fruit production or fruit set. Our results, thus, indicate that plants do not respond to damage by adjusting fruiting probability of flowering plants or fruit set. Fruit setting may contribute to the costs of sexual reproduction but, at least according to our results, at a similar level in all experimental groups.

The negative effects of herbivory are supposed to be more pronounced when plants are developing reproductive structures that are related to sexual reproduction (e.g., Tiffin, 2000). In accordance with this, our results showed that while herbivore damage had negative effects on sexual reproduction, no effects at all were found on clonal reproduction. This might be due to the allocation trade‐off between sexual and clonal reproduction. Allocation trade‐offs can be either resource‐based or more direct when clonal organs replace sexual structures, and they are likely to limit the fitness gain through either reproductive mode (Ronsheim & Bever, 2000; Vallejo‐Marín, Dorken, & Barrett, 2010). However, allocation trade‐offs have only rarely been detected at the level of genet (Vallejo‐Marín et al., 2010). Whether or not allocation trade‐offs exist, clonal reproduction is often associated with an increase in the size of individual plants and, thus, it is likely to help the plant to acquire more resources (Vallejo‐Marín et al., 2010). High resource intake is likely to be advantageous when compensating for herbivore damage. On the other hand, sexual reproduction is known to promote adaptations by increasing and maintaining genetic variation through recombination. Plant defences are usually genetically determined traits showing heritable variation and are often under selection exerted by herbivores (Berenbaum, Zangerl, & Nitao, 1986; Mauricio, 1998; Juenger, Lennartsson, & Tuomi, 2000; Baucom & Mauricio, 2004). Sexual reproduction can, thus, be considered beneficial to plants exposed to herbivory (Hamilton et al., 1990; Johnson et al., 2009). Our results showed that although only a small proportion of the experimental plants flowered and set fruits during the first year, even some of the plant individuals that experienced the largest amount of experimental damage (50%) did so. This might indicate that even when facing severe defoliation plants maintain at least minimal levels of sexual reproduction in order to ensure the benefits of sexuality. Further studies are needed to understand the roles of sexual and asexual reproduction when plants are facing herbivory, and whether the patterns observed in this study are due to resource allocation trade‐offs between the different modes of reproduction.

In the year following damage, experimental plants growing in the common garden were damaged by herbivores present at the site. The highest amount of natural leaf damage during the second year was around 20% leaf area consumed. We found a positive correlation between natural herbivory and the number of flowers, indicating that herbivores preferred more vigorously growing plants (Price, 1991). The same phenomenon has also been found in other plant species (Haysom & Coulson, 1998; Neuvonen & Niemelä, 1981; Schlinkert et al., 2015). Plants that experienced more experimental damage produced, on average, fewer flowers during the following year. If herbivores do prefer vigorous woodland strawberries, this finding could indicate that plants suffering the negative effects of herbivory during subsequent years may experience less damage, and, thus, have more resources to allocate for compensation.

## 5 CONCLUSIONS

The results of this study show how the negative effects of folivory depend not only on the amount of damage but also on the time frame used to study the effects. This is likely to be because of differences in resource allocation after herbivore damage. This problematizes our view of tolerance—what appears to be tolerance in 1 year actually turns out to be a cost that is deferred in time. Our results, thus, underline the importance of studying the effects of herbivore damage over several consecutive years in perennial plants. In addition, our results show how negative effects of herbivory are consistently channeled to sexual reproduction, while clonal reproduction remains unaffected. Given that both modes of reproduction usually have different ecological and evolutionary consequences for different aspects of plant life, it is important to consider the role of both these modes when studying the effects of herbivory.

## CONFLICT OF INTEREST

Both authors declare that the research was conducted in the absence of any commercial or financial relationships that could be construed as a potential conflict of interest.

## AUTHOR CONTRIBUTIONS

Both authors conceived the ideas, designed methodology, and collected the data; AM analyzed the data; AM led the writing of the manuscript. Both authors contributed critically to the drafts and gave final approval for publication.

## DATA ACCESSIBILITY

Data will be stored at the Dryad Digital Repository (https://doi.org/10.5061/dryad.69r593t).
